# High-Throughput Chinmedomics Strategy Discovers the Quality Markers and Mechanisms of Wutou Decoction Therapeutic for Rheumatoid Arthritis

**DOI:** 10.3389/fphar.2022.854087

**Published:** 2022-04-12

**Authors:** Taiping Li, Fangfang Wu, Aihua Zhang, Hui Dong, Ihsan Ullah, Hao Lin, Jianhua Miao, Hui Sun, Ying Han, Yanmei He, Xijun Wang

**Affiliations:** ^1^ National Engineering Laboratory for the Development of Southwestern Endangered Medicinal Materials, Guangxi Botanical Garden of Medicinal Plant, Nanning, China; ^2^ National Chinmedomics Research Center, National TCM Key Laboratory of Serum Pharmacochemistry, Metabolomics Laboratory, Heilongjiang University of Chinese Medicine, Harbin, China

**Keywords:** metabolomics, mass spectrometry, biomarker, pathway, quality markers, chinmedomics, effective subustance

## Abstract

Wutou decoction (WTD) is a traditional Chinese medicine prescription for the treatment of rheumatoid arthritis (RA), and this study systematically analyzed the metabolic mechanism and key pharmacodynamic components of WTD in RA rats by combining untargeted metabolomics and serum pharmacochemistry of traditional Chinese medicine to enrich the evidence of WTD quality markers (Q-markers) studies. WTD prevented synovial edema in RA rats and reduced tumor necrosis factor-alpha and interleukin 6 levels in rat serum, according to the results of an enzyme-linked immunosorbent examination and histopathological inspection. In model rats, pattern recognition and multivariate statistical analysis revealed 24 aberrant metabolites that disrupted linoleic acid metabolism, arachidonic acid metabolism, arginine and proline metabolism, *etc*. However, continued dosing of WTD for 28 days reversed 13 abnormal metabolites, which may be an important therapeutic mechanism from a metabolomic perspective. Importantly, 12 prototypical components and 16 metabolites from WTD were characterized in RA rat serum. The results of Pearson correlation analysis showed that aconitine, L-ephedrine, L-methylephedrine, quercetin, albiflorin, paeoniflorigenone, astragaline A, astragaloside II, glycyrrhetic acid, glycyrrhizic acid, licurazide, and isoliquiritigenin are the key pharmacological components that regulate the metabolism of RA rats, and they are identified as Q-markers. In sum, utilizing metabolomics and serum pharmacochemistry of traditional Chinese medicine, the metabolic mechanisms and Q-markers of WTD therapy in RA rats were revealed, providing a theoretical basis for the quality control investigation of WTD.

## Introduction

Traditional Chinese medicine has a long history of application and accumulated rich clinical experience, TCM plays a pivotal role in the protection of human health, of which herbal medicine is the key medium. A large number of chemicals in herbs and prescriptions poses a difficult problem for quality control. Unlike in the past, the quality control of herbal medicines is not only concerned with the high content of the components in the herbal medicines, but the remarkable medicinal effects are more noticeable. The concept and requirements of quality markers (Q-markers) proposed by Academician Liu have promoted the development of quality control systems for TCM and herbal medicines. In general, ideal herbal medicines and prescriptions need to have five characteristics: *1*) quality transfer and traceability, *2*) component specificity, *3*) component effectiveness, *4*) component measurability, *5*) formula compatibility environment (He et al., 2018; Bai et al., 2018; Zhang et al., 2018).

Chinmedomics comprehensively blends metabolomics and serum pharmacochemistry of TCM and serves as a way for the discovery of Q-markers for herbal medicines and formulations (Zhang et al., 2019). In short, the metabolic mechanisms of herbal medicines and prescriptions for the treatment of diseases are gained through metabolomics insights (Zhang et al., 2017; Zhang et al., 2020; Qiu et al., 2020), and key pharmacodynamic components and Q-markers that regulate abnormal metabolism are identified from a large number of chemical components by correlation analysis (Wang et al., 2015; Han et al., 2020). Currently, chinmedomics was used as a research method for Q-markers to analyze the active components and therapeutic mechanisms of Shengmai San, Yinchenhao Decoction, Sijunzi Decoction, and Kaixin San (Zhang et al., 2018; Sun et al., 2019; Wang et al., 2019; Zhao et al., 2020).

**FIGURE 1:**
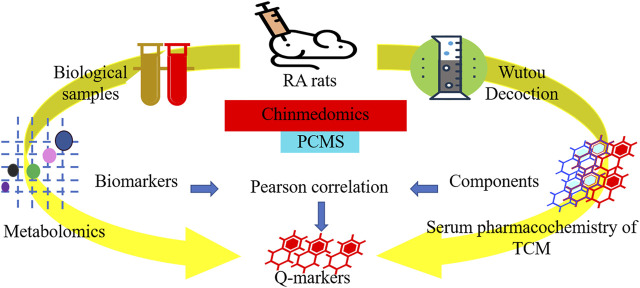
The workflow of quality markers research for wutou decoction in the treatment of adjuvant-induced arthritis rat based on chinmedomics strategy.

Wutou decoction (WTD) is a classic prescription for treatment rheumatology, derived from “*Shang Han Lun*” of Zhang Zhongjing in the Han Dynasty and consisting of *Aconitum carmichaeli* Debeaux [Ranunculaceae], *Ephedra sinica* Stapf [Ephedraceae], *Paeonia lactiflora* Pall. [Ranunculaceae], *Astragalus mongholicus Bunge* [Leguminosae], and *Glycyrrhiza uralensis* Fisch. [Leguminosae] with a mass ratio of 10 g: 15 g: 15 g: 15 g: 15 g. Pharmacological studies have shown WTD analgesic and anti-inflammatory effects (Guo et al., 2017; Guo et al., 2020). Besides, WTD mainly contains alkaloids, triamcinolone, and flavonoid compounds that can regulate the biological signal pathways associated with rheumatoid arthritis (RA) (Qi et al., 2014; Xu et al., 2017). To enhance the Q-markers and quality control evidence of WTD, this study adopted metabolomics and serum pharmacochemistry of the TCM to carefully evaluate the global metabolic profile of WTD-treated RA rats and the components of WTD that are closely related to the therapeutic effect (Figure 1).

## Material and Methods

### Reagents and Medicines

HPLC grade methanol and acetonitrile were purchased from Thermo Fisher Scientific (Massachusetts, United States), analytical grade formic acid, leucine enkephalin, and Freund’s complete adjuvant were purchased from Sigma-Aldrich (Shanghai, China), ultrapure water purchased from Watsons (Guangzhou, China), the kit of tumor necrosis factor-alpha (TNF-α) and interleukin-6 (IL-6) purchased from Jiancheng Biologics (Nanjing, China), the five botanical drugs that compose WTD were purchased from Tongrentang Pharmacy (Harbin, China) and were authenticated by Xijun Wang from the Department of Pharmacognosy of Heilongjiang University of Chinese Medicine.

### Preparation of Wutou Decoction Extracts

The crude *Aconitum carmichaeli* Debeaux [Ranunculaceae], *Ephedra sinica* Stapf [Ephedraceae], *Paeonia lactiflora* Pall. [Ranunculaceae], *Astragalus mongholicus* Bunge [Leguminosae], and *Glycyrrhiza uralensis* Fisch. [Leguminosae] cut into small round pieces according to the mass ratio 2: 3: 3: 3: 3 and were immersed in distilled water (eight-fold volume) for 1 h and then boiled for 1.5 h. The extracted solutions were filtered through eight layers of gauze, and the filter residue was boiled with distilled water (eight-fold volume) again for another 1 h. Finally, the two parts of the filtrates were combined, and the decoction was transformed into powder by freeze-drying. The frozen powder was dissolved with distilled water and kept at 4°C until use.

### Animal Grouping and Administration

Experiments were performed with eight-week-old male Sprague-Dawley rats weighing 180–220 g (Liaoning Changsheng Biotechnology Co., Ltd., License No. SCXK [Liao] 2015-0001) under controlled environmental conditions (12/12 light/dark, 23–25°C, 50 ± 5% humidity) with unlimited access to food and water. After 7 days of acclimatization, all animals were randomly divided into the following three groups (*n* = 8): control group (C); model group (M), and WTD treatment group (T). The M and T rats were injected with 0.1 ml of Freund’s complete adjuvant at a concentration of 2.0 mg.ml^−1^ at the base of the tail, and the C rats were injected with the same volume of normal saline (Zhang et al., 2014). On the 14th day of model preparation, rats in the T were orally administered a 7.56 g.kg^−1^ lyophilized powder solution of WTD once a day for 28 consecutive days (Dong et al., 2015). The C rats received the same volume of ultrapure water. The protocol was approved by the Committee on the Ethics of Animal Experiments of the College of Pharmacy of the Heilongjiang University of Chinese Medicine.

### Collect and Prepare Biological Samples

Throughout the experimental period, 12 h urine from 8 p.m. to 8 a.m. the next day was collected in the metabolic cage, and the blood samples were obtained by the final stage of administration: fresh urine centrifugation for 15 min (4°C, 13,000 r·min^−1^), serum centrifugation for 15 min (4°C, 3500 r·min^−1^). Urine samples were diluted 10 times with ultrapure water, serum samples were precipitated with four times the volume of methanol and the supernatant was taken. All samples were filtered through a 0.22 μm filter before injection into the ultra-performance liquid chromatography quadrupole time of flight coupled with mass spectrometry (UPLC-Q/TOF-MS) instrument. Separately, 100 μl of urine and serum were obtained from each animal for the preparation of quality control samples in examining and optimizing the metabolomics analysis parameters.

### Inflammatory Factors and Histopathological Examination

The concentrations of IL-6 and TNF- in serum were determined using the kit’s instructions. X-rays of the rat feet were taken to evaluate the effects of WTD. Fresh ankles were immersed in the formalin solution, and pathological changes were observed by the affiliated hospital of the Heilongjiang University of Chinese Medicine under proper microscopic circumstances.

### Metabolomic Conditions

#### Chromatography

The metabolomic analysis was performed using an ACQUITY UPLC^®^ system coupled with a time-of-flight mass spectrometer (Waters Corp.). The HSS T_3_ column (2.1 mm × 100 mm id, 1.8 µm; Waters Corp.) separates urine samples, whereas the BEH C_18_ column (2.1 mm × 100 mm id, 1.7 µm, Waters Corp.) separates serum samples. The column temperature is 35°C. The gradient mobile phase consisted of solvent A (0.1% FA-H_2_O) and solvent B (0.1% FA-ACN). Optimized urine gradient elution status: 0–4.5 min, 1–21 A%; 4.5–7 min, 21–40% A; 7–7.5 min, 40–50% A; 7.5–9.5 min, 50–99% A. Optimized serum gradient elution status: 0–1.2 min, 1–39% A; 1.2–4.3 min, 39–68% A; 4.3–4.5 min, 68–71% A; 4.5–7.0 min, 71–78% A; 7.0–8.2 min, 78–90% A; 8.2–9.5 min, 90–99% A. The injection volume is 4 μl and the flow rate is 0.5 ml.min^−1^ in both modes.

#### Mass Spectrometric

The Waters G2-Si mass spectrometer system (Waters Corp.) is equipped with an electrospray ion source to detect all metabolites, and the ion source temperature is 110°C. The capillary voltage in the positive ion mode (ESI^+^) is 3.0 kV; in the negative ion mode (ESI^−^), it is 2.7 kV. The cone voltage is 20 V, and the gas flow rate is 50 L.h^−1^. Dry nitrogen is desolventizing at a flow rate of 800 L.h^−1^ and a temperature of 350°C. Use the full scan mode of m/z 50–1200 Da to collect MS data. Leucine enkephalin with a concentration of 1 ng/μl and a flow rate of 10 μl.min^−1^ in ESI^+^ [M+H]^+^ = 556.2771 and ESI^−^ [M-H]^−^ = 554.2615 was used as a reference compound to obtain an accurate mass.

### Constituent Analysis of Wutou Decoction

After lyophilized powder of WTD decoction was dissolved in ultrapure water and was centrifuged 15 min (4°C, 13,000 r·min^−1^), filtered with a 0.22 μm filter membrane, and injected into UPLC-MS for analysis chemical profile of WTD. The serum containing components of WTD was added with a quarter volume of 20% phosphoric acid, utilizing an OASIS MCX solid-phase extraction column (30 mg, 30 μm, Waters Corp.) to enrich the WTD components *in vivo*, dissolved in methanol for UPLC-MS data collection. Injection volume is 2 μl, the gradient mobile phase consisted of solvent A (0.1% FA-H_2_O) and solvent B (0.1% FA-ACN), optimized gradient elution conditions: 0–3.0 min, 1–10% A; 3.0–7.0 min, 10–12% A; 12.0–20.0 min, 20–40% A; 20.0–22.0 min, 40–100% A. The MS^E^ data scanning mode of UPLC-Q/TOF-MS was combined with powerful UNIFI software (Waters Corp.) for efficient and accurate characterization of the chemical profile of the WTD and the components absorbed *in vivo*. The adduct ion is [M+H]^+^ in ESI^+^ and [M-H]^-^ in ESI^−^, respectively. To reduce false positives, select the embedded TCM database and set the tolerance error of components and fragments to 5 mDa. The study compared the serum between the M and the T and preferred the results of the high response value only in the serum of the T.

### Multivariate Statistics and Data Analysis

The raw data is imported into Progenesis QI software (Waters Corp.) for noise reduction, peak alignment, and normalization to get an information matrix that includes m/z, retention time, and peak relative intensity. The EZinfo software (Waters Corp.) performs a principal component analysis (PCA) and judges differences between groups. Orthogonal partial least squares discriminant analysis (OPLS-DA) calculates variable importance in projection value (VIP), a statistical normalization abundance of different ions. Finally, MS/MS information combined with HMDB (https://hmdb.ca/) and MassBank (http://www.massbank.jp/) databases identifies metabolites, VIP >1, and metabolites normalized abundance with intergroup significance are considered potential biomarkers of RA rats. Comparisons between the two groups of the quantitative data were completed using Graphpad software (California, United States) independent *t*-test and presented as mean ± standard deviation, with *p* < 0.05 indicating significant differences and *p* < 0.01 indicating extremely significant differences.

### Analysis of Key Medicinal Constituents

The PCMS (plotting of correlation between marker metabolites and serum components) patented software calculates the correlation coefficient between the components absorbed into the blood of WTD and the potential biomarkers of RA rats to determine which component is most likely to have a therapeutic effect. Normalize the abundance of biomarkers and components, and then use Pearson’s statistical correlation to set the reference standard for the correlation coefficient R. In this study, 0.7 ≤│R│<0.8 is selected as the highly correlated component; 0.8 ≤│R│≤ 1 is regarded as extremely correlated, and extremely correlated components are regarded as Q-markers.

## Results

### Therapeutic Effects of Wutou Decoction

**FIGURE 2:**
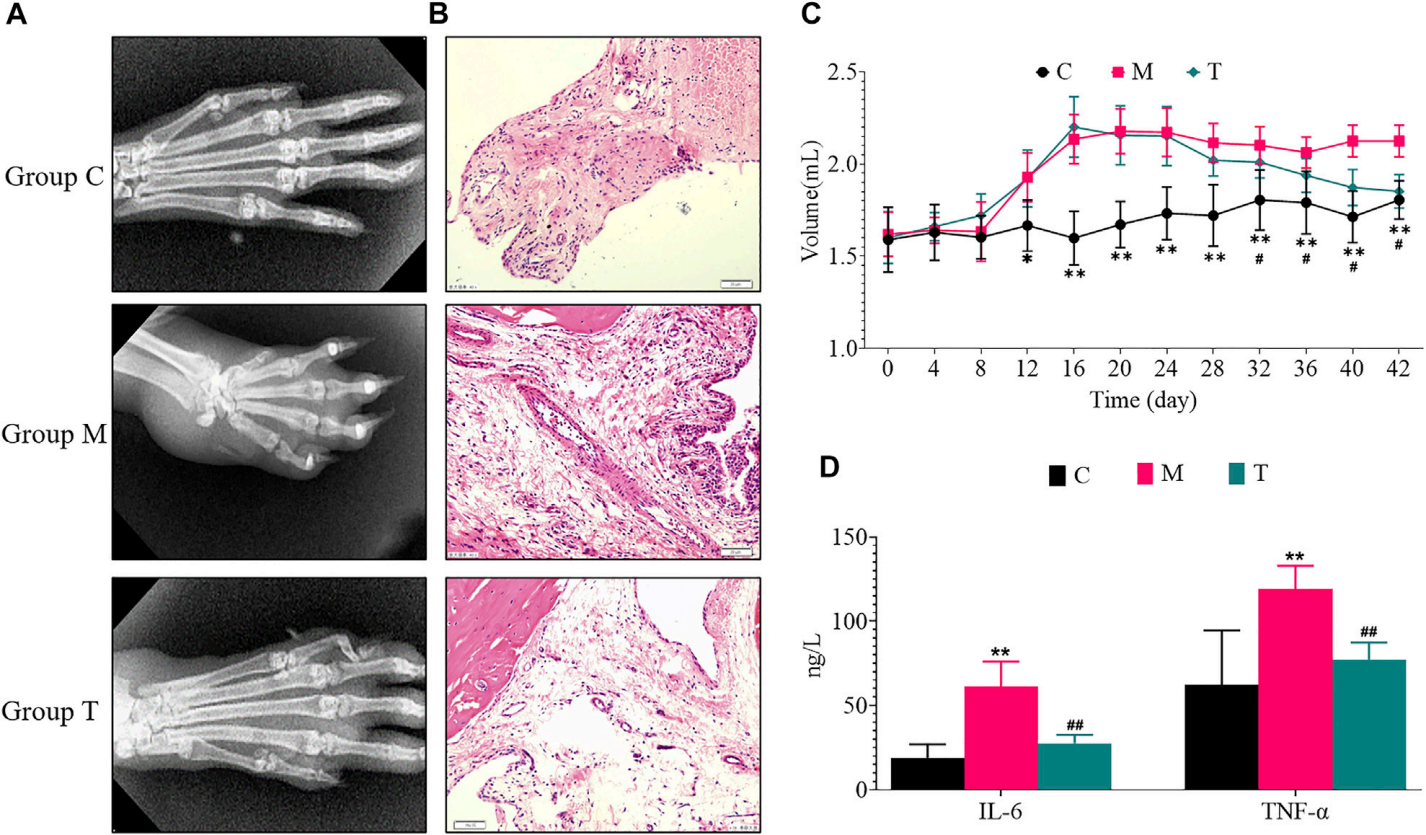
Evaluation of the effect of wutou decoction on adjuvant-induced arthritis rat. **(A)** X-ray examination **(B)** Synovial histopathological examination (*200) **(C)** changes in paw volume before and after administration **(D)** Determination of inflammatory factors in serum. Results are expressed as mean ± SD, *n* = 8. ^*^ *p* < 0.05, ^**^ *p* < 0.01 vs control group. ^#^ *p* < 0.05, ^##^ *p* < 0.01 vs model group.

Compared with the C rats, 14 days after the injection of Freund’s complete adjuvant, the rats in the M reached the uttermost state of inflammation. Compared with the M, the T significantly inhibited the levels of IL-6 and TNF-α in rats and reduced the swelling of the feet (Figure 2).

### Characterize Potential Biomarkers

**FIGURE 3:**
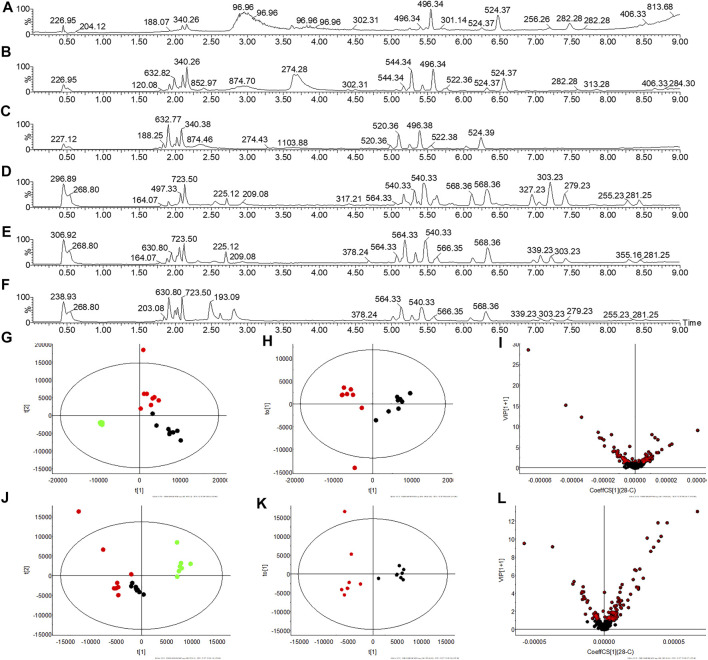
Serum metabolic profile collection and multivariate statistical analysis. **(A**–**C)** TIC spectra of C, M, and T rats in ESI^+^, respectively **(D**–**F)** TIC spectra of C, M, and T rats in ESI^−^, respectively **(G**,**J)** PCA plot of C, M, and T in ESI^+^ and ESI^−^, respectively **(H**,**K)** OPLS-DA plot of C and M in ESI^+^ and ESI^−^, respectively **(I**,**L)** VIP plot of C and M in ESI^+^ and ESI^−^, respectively. Note: 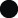: C rats; 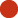: M rats; 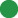: T rats.

**FIGURE 4:**
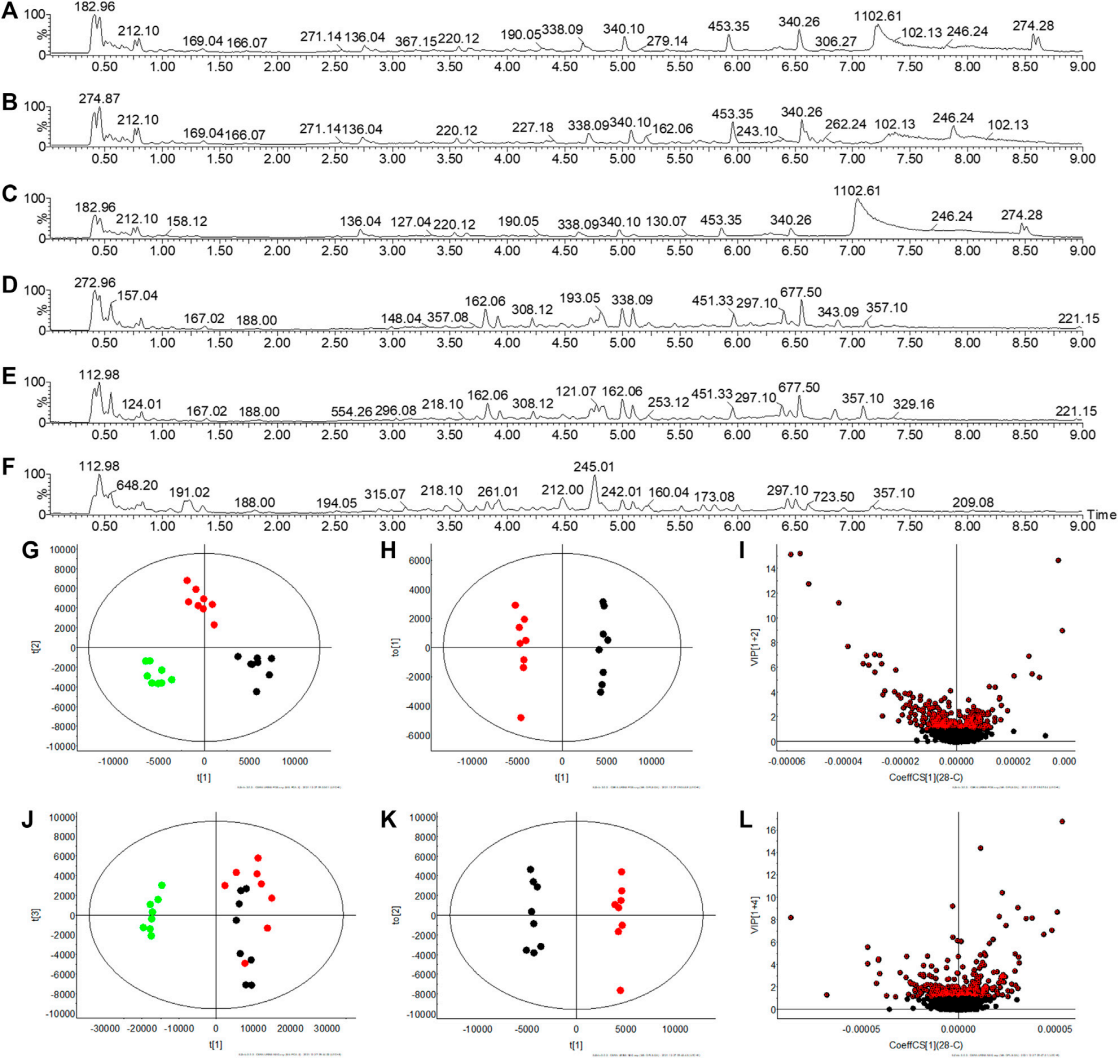
Urine metabolic profile collection and multivariate statistical analysis. **(A**–**C)** TIC spectra of C, M, and T rats in ESI^+^, respectively **(D**–**F)** TIC spectra of C, M, and T rats in ESI^−^, respectively **(G**,**J)** PCA plot of C, M, and T in ESI^+^ and ESI^−^, respectively **(H**,**K)** OPLS-DA plot of C and M in ESI^+^ and ESI^−^, respectively **(I**,**L)** VIP plot of C and M in ESI^+^ and ESI^−^, respectively. Note: 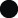: C rats; 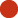: M rats; 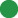: T rats.

Multivariate statistical analysis was performed on the metabolic fingerprint information collected from serum and urine at the end of the experiment. There are differences in serum total ion chromatograms (TIC) in C, M, and T rats in ESI^+^ and ESI^−^ and these are reflected in PCA (Figure 3). Similarly, there are differences in the TIC of the urine between the three groups, suggesting a change in the metabolic profile (Figure 4). The OPLS-DA statistical model calculates the VIP of differential ions and is employed to screen potential biomarkers, the R2Y and Q2 values of the urine model (R2Y (cum) = 0.995, Q2(cum) = 0.927 in ESI^+^, and R2Y(cum) = 0.994, Q2(cum) = 0.615 in ESI^−^) and serum model (R2Y (cum) = 0.858, Q2(cum) = 0.798 in ESI^+^, and R2Y (cum) = 0.872, Q2(cum) = 0.795 in ESI^−^) indicated that the statistic model has good quality and predictability.

Take arachidonic acid as an example to illustrate the identification process of potential biomarkers. First, OPLS-DA calculated that the VIP of 8.09_303.2320m/z was 4.004 and the normalized abundance of this ion in the serum of the C and the M was different (*p* < 0.05). Secondly, chromatographic peak extraction and elemental composition analysis showed that the tolerance of C_20_H_32_O_2_ was accepted, while m/z 304 and m/z 279 were simultaneously observed in MS extraction at 8.09 min. Finally, it was identified that m/z 304 Da is the parent ion of arachidonic acid, and the fragments of 303[M-H]^−^ and 279[M-COOH]^-^ were generated by high-energy bombardment in ESI^−^ (Supplementary Figure S1). Eventually, 24 biomarkers are identified (Table 1).

**TABLE 1:** Identification results of potential biomarker groups in adjuvant-induced arthritis rats.

No	Compound	Adducts	Formula	R_t_ (min)	m/z	Mass error (ppm)	MS/MS	Description	HMDB ID	VIP value
1	0.48_175.1195m/z	M+H	C_6_H_14_N_4_O_2_	0.48	175.1195	3.37	M1:158[M+H-OH]^+^	L-Arginine	HMDB0000517	1.0852
							M2:130[M+H-COOH]^+^			
							M3:116[M-NC(NH_2_)2]^+^			
2	0.52_204.1238m/z	M+H	C_9_H_17_NO_4_	0.52	204.1238	3.73	M1:144[M-N(CH_3_)3]^+^	L-Acetylcarnitine	HMDB0000201	3.0411
							M2:129[M1-CH_3_]^+^			
							M3:85[M1-CH_3_COO]^+^			
3	3.51_852.6867m/z	M+Na	C_48_H_96_NO_7_P	3.51	852.6867	6.13	M1:829[M]^+^	PC(22:0/P-18:0)	HMDB0008555	1.1812
							M2:184[M-C_43_H_83_O_3_+2H]^+^			
							M3:86[M-C_43_H_83_O_7_P]^+^			
4	4.01_542.3282m/z	M+H	C_28_H_48_NO_7_P	4.01	542.3282	7.58	M1:523[M-H_2_O]^+^	LysoPC(20:5(5Z,8Z,11Z,14Z,17Z))	HMDB0010397	1.1387
							M2:104[M-C_23_H_35_O_6_P+H]^+^			
							M3:86[M-C_23_H_35_O_7_P]^+^			
5	4.99_424.3425m/z	M+H	C_25_H_45_NO_4_	4.99	424.3425	0.92	M1:365[M+H-N(CH_3_)3]^+^	Linoleyl carnitine	HMDB0006469	2.3001
							M2:85[M1-C_17_H_31_COO]^+^			
							M3:71[M2-CH_2_]^+^			
							M4:57[M3-CH_2_]^+^			
6	5.12_400.3446m/z	M+H	C_23_H_45_NO_4_	5.12	400.3446	6.21	M1:341[M-CH_2_COO]^+^	L-Palmitoylcarnitine	HMDB0000222	1.7873
							M2:85[M-N(CH_3_)3-C_15_H_31_COO]^+^			
							M3:71[M2-CH_2_]^+^			
7	5.25_426.3583m/z	M+H	C_25_H_47_NO_4_	5.25	426.3583	1.17	M1:426[M+H]^+^	Octadecenoylcarnitine	HMDB0094687	2.9946
							M2:85[M-C_21_H_43_NO_2_]^+^			
8	6.39_481.2336m/z	M+Na	C_23_H_39_O_7_P	6.39	481.2336	2.29	M1:481[M+Na]^+^	LPA(0:0/20:4n6)	HMDB0012496	1.1573
							M2:361[M-C_23_H_37_O_3_]^+^			
							M3:287[M-C_19_H_31_CO]^+^			
							M4:185[M+2H-OH-C_19_H_31_]^+^			
							M5:133[M+H-H_2_PO_3_-C_18_H_29_]^+^			
9	4.69_378.2401m/z	M-H	C_18_H_38_NO_5_P	4.69	378.2401	−3.54	M1:379[M]^−^	Sphingosine 1-phosphate	HMDB0000277	5.0357
							M2:378[M-H]^−^			
							M3:78[M-OH-C_18_H_37_NO]^−^			
10	8.09_303.2320m/z	M-H	C_20_H_32_O_2_	8.09	303.2320	−3.27	M1:304[M]^−^	Arachidonic acid	HMDB0001043	4.0040
							M2:303[M-H]^−^			
							M3:259[M-COOH]^−^			
11	5.12_317.2110m/z	M-H	C_20_H_30_O_3_	5.12	317.2110	−3.78	M1:317[M-H]^−^	15-HEPE	HMDB0010209	1.3047
							M2:299[M1-H_2_O]^−^			
12	5.45_452.2775m/z	M-H	C_21_H_44_NO_7_P	5.45	452.2775	−1.76	M1:452[M-H]^−^	LysoPE(0:0/16:0)	HMDB0011473	2.3420
13	5.06_279.2316m/z	M-H	C_18_H_32_O_2_	5.06	279.2316	−4.70	M1:280[M]^−^	Linoleic acid	HMDB0000673	3.1980
							M2:279[M-H]^−^			
							M3:261[M2-H_2_O]^−^			
14	0.65_335.0966m/z	M+Na	C_11_H_20_O_10_	0.65	335.0966	5.60	M1:335[M+Na]^+^	Galactose-beta-1,4-xylose	HMDB0011677	1.0580
							M2:145[M-CH_2_OH-C_5_H_9_O_5_]^+^			
15	0.49_103.0029m/z	M-H	C_3_H_4_O_4_	0.49	103.0029	−7.93	M1:104[M]^−^	Hydroxypyruvic acid	HMDB0001352	2.0564
							M2:56[M-CH_2_OH-OH]^−^			
16	0.51_209.0290m/z	M-H	C_6_H_10_O_8_	0.51	209.0290	−6.26	M1:209[M-H]^−^	Glucaric acid	HMDB0000663	1.0750
							M2:165[M-COOH]^−^			
							M3:103[M-OH-(COOH)2]^−^			
							M4:75[M-(CHOH)3COOH]^−^			
17	0.56_212.0219m/z	M+FA-H	C_4_H_9_NO_4_S	0.56	212.0219	−9.27	M1:166[M-H]^−^	N-Acetyltaurine	HMDB0240253	1.9768
							M2:124[M-COCH_3_]^−^			
							M3:80[M-H-(CH_2_)2NHCOCH_3_]^−^			
							M4:58[M-(CH_2_)2SO_3_H]^−^			
18	0.60_179.0549m/z	M-H	C_6_H_12_O_6_	0.60	179.0549	−6.55	M1:179[M-H]^−^	D-Glucose	HMDB0000122	1.2602
							M2:161[M1-H_2_O]^−^			
							M3:143[M2-H_2_O]^−^			
							M4:125[M3-H_2_O]^−^			
							M5:59[M4-CHOCH_2_OH]^−^			
19	1.85_131.0818m/z	M-H	C_5_H_12_N_2_O_2_	1.85	131.0818	−5.77	M1:131[M-H]^−^	Ornithine	HMDB0000214	1.8019
							M2:99[M1-(NH_2_)2]^−^			
							M3:67[M2-H_2_O-CH_2_]^−^			
20	3.28_178.0499m/z	M-H	C_9_H_9_NO_3_	3.28	178.0499	−6.01	M1:178[M-H]^−^	Hippuric acid	HMDB0000714	3.3685
							M2:134[M-COOH]^−^			
							M3:77[M-CONHCH_2_COOH]^−^			
							M4:59[M-C_6_H_5_CONH]^−^			
21	4.43_188.0345m/z	M-H	C_10_H_7_NO_3_	4.43	188.0345	−4.29	M1:188[M-H]^−^	Kynurenic acid	HMDB0000715	1.6490
							M2:144[M-COOH]^−^			
22	5.52_201.1120m/z	M+FA-H	C_9_H_16_O_2_	5.52	201.1120	−7.89	M1:201[M+FA-H]^−^	4-Hydroxynonenal	HMDB0004362	1.0608
							M2:121[M-OH-H_2_O]^−^			
23	7.58_349.2004m/z	M-H	C_20_H_30_O_5_	7.58	349.2004	−4.84	M1:349[M-H]^−^	Prostaglandin E3	HMDB0002664	1.7470
							M2:331[M1-H_2_O]^−^			
							M3:313[M2-H_2_O]^−^			
							M4:249[M-H-H_2_O-CHCH_2_C_4_H_7_]^−^			
24	8.78_331.1911m/z	M+FA-H	C_19_H_26_O_2_	8.78	331.1911	−1.38	M1:285[M-H]^−^	Androstenedione	HMDB0000053	1.6056
							M2:225[M-C_2_H_5_O_2_]^−^			

### Enrichment Analysis of Potential Biomarkers

**FIGURE 5:**
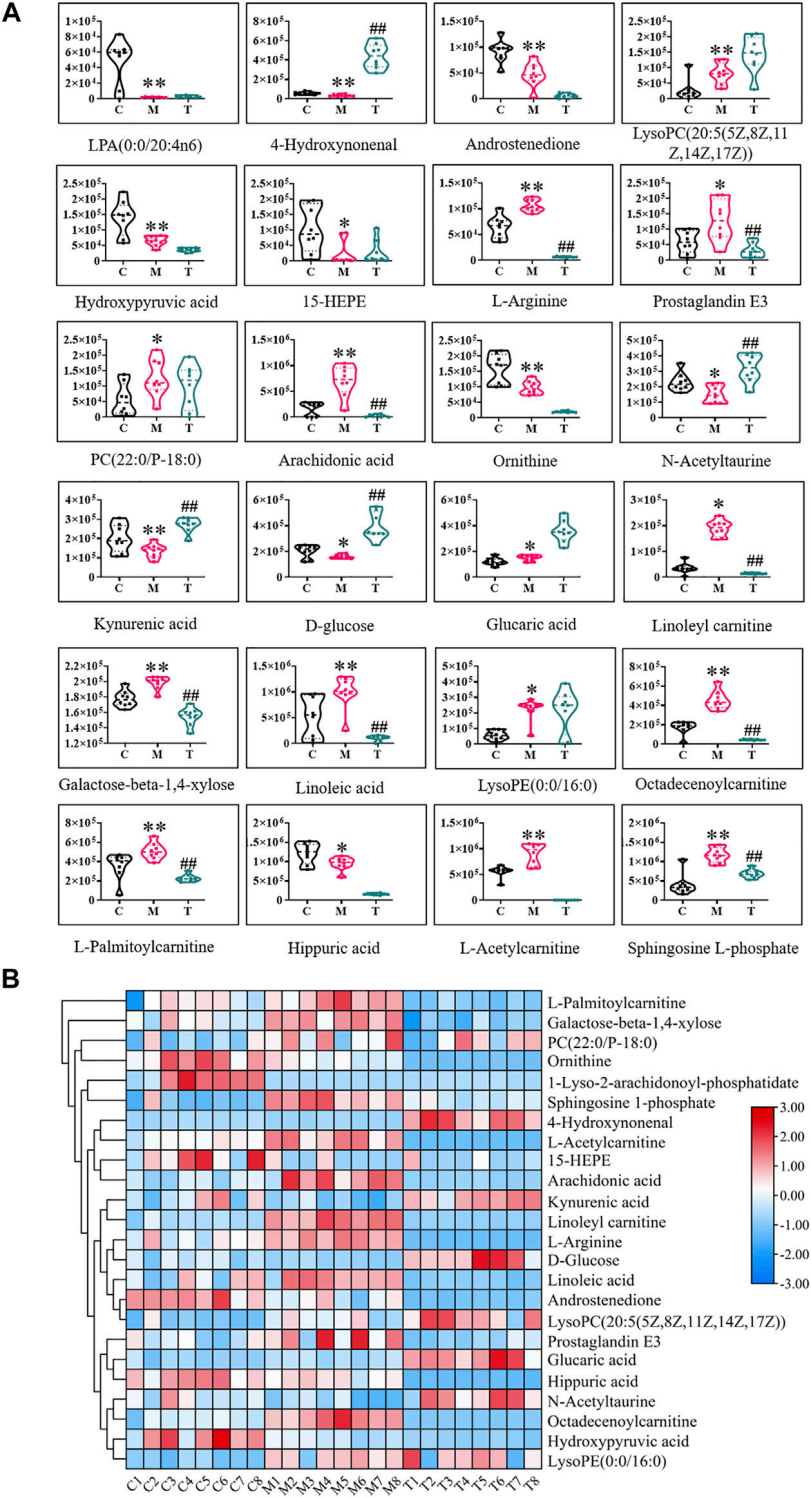
The normalized abundance of potential biomarkers before and after wutou decoction administration. **(A)** Normalized abundance values between different groups **(B)** Heat map of the differences in the potential biomarkers of the C, M and T obtained in the ESI^+^ and ESI^−^. Results are expressed as mean ± SD, *n* = 8. ^*^ *p* < 0.05, ^**^ *p* < 0.01 vs control group. ^#^ *p* < 0.05, ^##^ *p* < 0.01 vs model group. C represents control group, M represents model group, T represents wutou decoction group.

**FIGURE 6:**
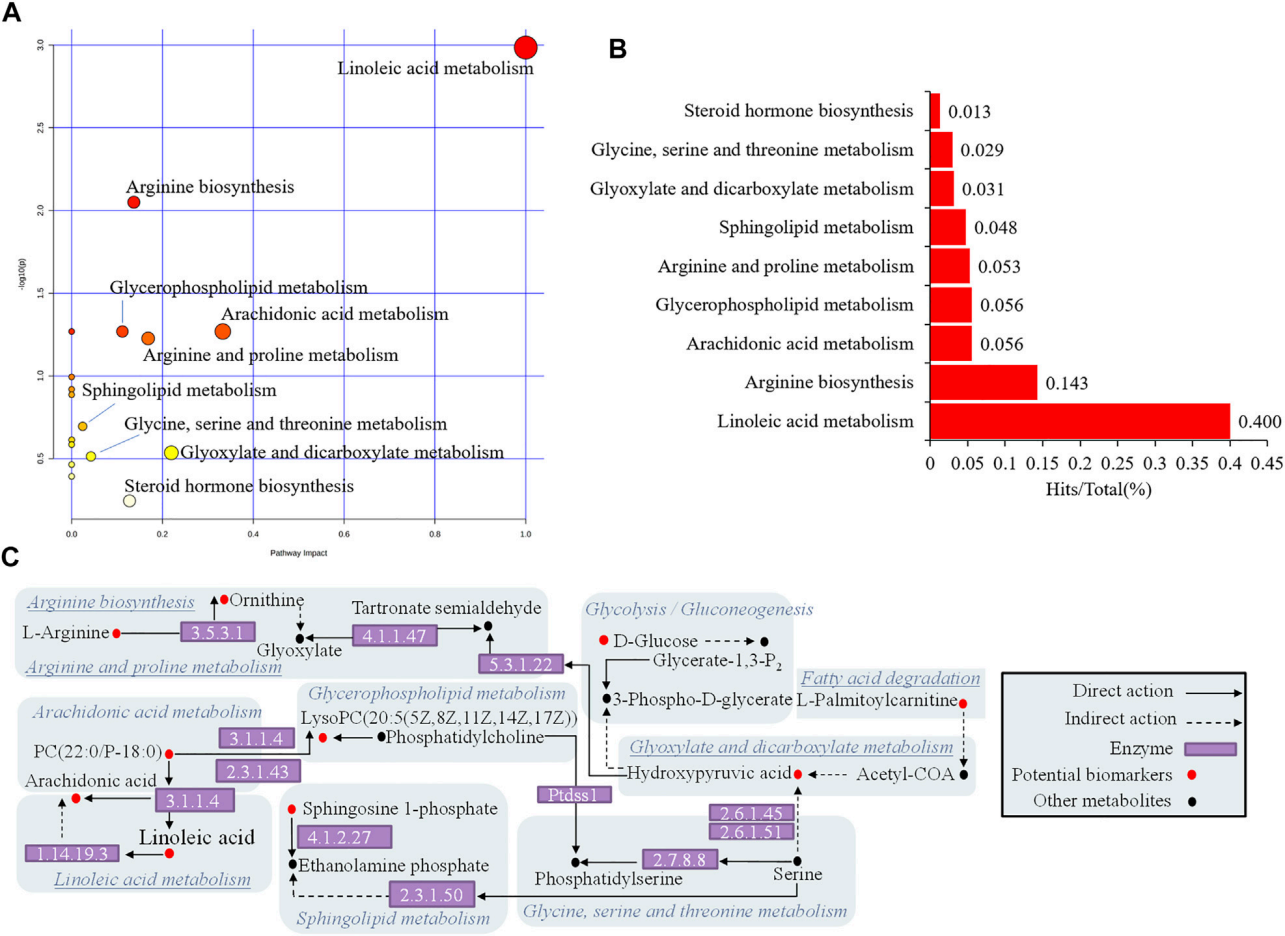
Enrichment analysis of metabolic pathway of potential biomarkers. **(A)** Pathway impact enrichment **(B)** The proportion of biomarkers in the overall pathway **(C)** Association network of biomarker metabolic pathways.

The above 24 potential biomarkers of serum and urine are mostly engaged in 17 metabolic pathways, which belong to lipid metabolism, carbohydrate metabolism, and amino acid metabolism (Table 2). Where the impact value is greater than 0.1, linoleic acid metabolism, arachidonic acid metabolism, glyoxylate and dicarboxylate metabolism, arginine and proline metabolism, arginine biosynthesis, steroid hormone biosynthesis, and glycerophospholipid metabolism are considered important metabolic pathways. The WTD significantly reversed potential biomarkers with abnormal levels of 13/24 (Figure 5), including 4-Hydroxynonenal, L-Arginine, Prostaglandin E3, Aracidonic acid, N-Acetyltaurine, Kynurenic acid, D-Glucose, Linoleyl carnitine, Galactose-beta-1,4-xylose, Linoleic acid, Octadecenoylcarnitine, L-Palmitoylcarnitine, Sphingosine L-phosphate. WTD may correct the disturbed Linoleic acid metabolism, Arachidonic acid metabolism, Arginine and proline metabolism, Arginine biosynthesis, Steroid hormone biosynthesis, Sphingolipid metabolism, and Glycolysis/Gluconeogenesis by reversing the levels of the above markers, thereby treating Freund’s complete adjuvant-induced RA rats (Figure 6).

**TABLE 2:** Metabolic pathway analysis based on MetPA.

Pathway name	Class	Total	Expected	Hits	Raw p	−LOG_10_(p)	Holm adjust	FDR	Impact	Hits/Total	Potential biomarkers
Linoleic acid metabolism	Lipid metabolism	5	0.0530	2	0.0010	2.9850	0.0870	0.0870	1.0000	0.4000	PC(22:0/P-18:0), Linoleic acid
Arachidonic acid metabolism	Lipid metabolism	36	0.3817	2	0.0539	1.2688	1.0000	0.8310	0.3329	0.0556	PC(22:0/P-18:0), Arachidonic acid
Glyoxylate and dicarboxylate metabolism	Carbohydrate metabolism	32	0.3393	1	0.2916	0.5353	1.0000	1.0000	0.2196	0.0313	Hydroxypyruvic acid
Arginine and proline metabolism	Amino acid metabolism	38	0.4029	2	0.0594	1.2265	1.0000	0.8310	0.1685	0.0526	L-Arginine, Ornithine
Arginine biosynthesis	Amino acid metabolism	14	0.1484	2	0.0089	2.0502	0.7395	0.3742	0.1371	0.1429	L-Arginine, Ornithine
Steroid hormone biosynthesis	Lipid metabolism	77	0.8164	1	0.5693	0.2447	1.0000	1.0000	0.1275	0.0130	L-Palmitoylcarnitine
Glycerophospholipid metabolism	Lipid metabolism	36	0.3817	2	0.0539	1.2688	1.0000	0.8310	0.1118	0.0556	PC(22:0/P-18:0), LysoPC(20:5(5Z,8Z,11Z,14Z,17Z))
Glycine, serine and threonine metabolism	Amino acid metabolism	34	0.3605	1	0.3068	0.5131	1.0000	1.0000	0.0425	0.0294	Hydroxypyruvic acid
Sphingolipid metabolism	Lipid metabolism	21	0.2227	1	0.2018	0.6951	1.0000	1.0000	0.0243	0.0476	Sphingosine 1-phosphate
Glycolysis/Gluconeogenesis	Carbohydrate metabolism	26	0.2757	1	0.2438	0.6129	1.0000	1.0000	0.0002	0.0385	D-Glucose
Biosynthesis of unsaturated fatty acids	Lipid metabolism	36	0.3817	2	0.0539	1.2688	1.0000	0.8310	0.0000	0.0556	Linoleic acid, Arachidonic acid
Ascorbate and aldarate metabolism	Carbohydrate metabolism	10	0.1060	1	0.1014	0.9940	1.0000	1.0000	0.0000	0.1000	Glucaric acid
Phenylalanine metabolism	Amino acid metabolism	12	0.1272	1	0.1205	0.9191	1.0000	1.0000	0.0000	0.0833	Hippuric acid
alpha-Linolenic acid metabolism	Lipid metabolism	13	0.1378	1	0.1299	0.8864	1.0000	1.0000	0.0000	0.0769	PC(22:0/P-18:0)
Glutathione metabolism	Metabolism of other amino acids	28	0.2969	1	0.2601	0.5849	1.0000	1.0000	0.0000	0.0357	Ornithine
Fatty acid degradation	Lipid metabolism	39	0.4135	1	0.3437	0.4639	1.0000	1.0000	0.0000	0.0256	L-Palmitoylcarnitine
Aminoacyl-tRNA biosynthesis	Genetic Information Processing, Translation	48	0.5090	1	0.4054	0.3921	1.0000	1.0000	0.0000	0.0208	L-Arginine

### Blood Components of Wutou Decoction

**FIGURE 7:**
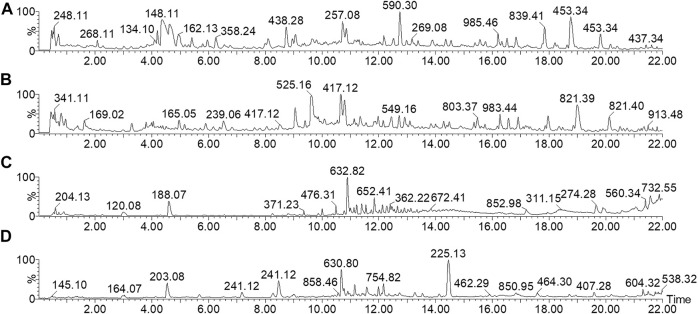
TIC spectrum of chemical profile and absorbed components from wutou decoction. **(A**,**B)** The chemical profile of wutou decoction in ESI^+^ and ESI^−^, respectively **(C**,**D)** The blood components of wutou decoction in ESI^+^ and ESI^−^, respectively.

The UPLC-Q/TOF-MS collects the TIC of WTD before and after entering the blood in ESI^+^ and ESI^−^ (Figure 7). The UNIFI software, with a powerful analysis module, can automatically identify the structure information obtained under high and low energy, including m/z, retention time, and MS/MS fragment response values, and deduce the cleavage relationship of compounds. Compound aconitine was used as an example to describe the process of applying UNIFI software to analyze the chemical profile of WTD. The UNIFI software matched the MS^E^ data collected in the continuum mode. Both ion response values between groups and all m/z information at 14.12 min were obtained, and m/z 646.32 Da with a high response value in WTD samples was scanned for small molecule ions. After elemental composition and fragment matching, m/z 646.32 Da was recognized as the hydrogenation ion of aconitine and identified to 586.30 Da [M+H-C_2_H_4_O_2_]^+^, 568.29 Da [M+H-C_2_H_6_O_3_]^+^, 554.27 Da [M+H-C_3_H_8_O_3_]^+^ (Supplementary Figure S2). Finally, they characterized and identified 61 components of the WTD (Supplementary Table S1). Importantly, 19 components were absorbed into the blood, including 12 prototype components (Supplementary Table S2).

### Discover the Key Medicinal Components

**FIGURE 8:**
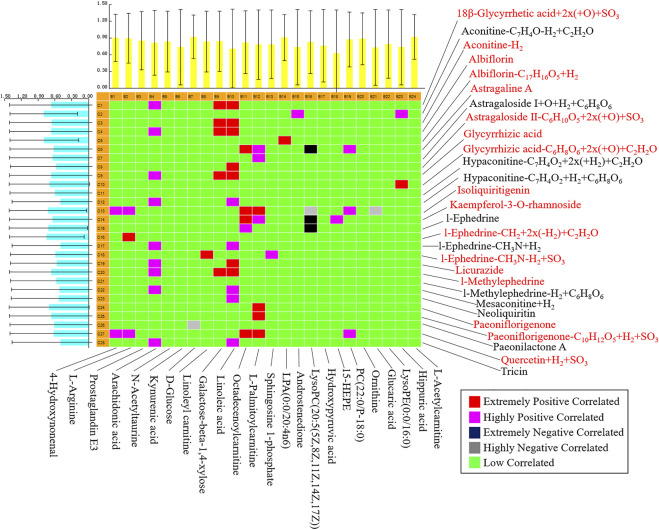
Pearson correlation analysis of blood components of wutou decoction and potential biomarkers in adjuvant-induced arthritis rat.

PCMS software was utilized in this investigation to uncover the important pharmacodynamic components of WTD in the treatment of RA rats, as well as biological activity tracking and literature analysis to identify Q-markers. The findings revealed that 19 of the WTD components were absorbed into the serum. According to correlation analysis, 12 components were confirmed as Q-markers (Figure 8), of which aconitine was from *Aconitum carmichaeli* Debeaux [Ranunculaceae], L-ephedrine, L-methylephedrine, quercetin, and kaempferol-3-O-rhamnoside were from *Ephedra sinica* Stapf [Ephedraceae], albiflorin and paeoniflorigenone were from *Paeonia lactiflora* Pall. [Ranunculaceae], astragaline A, and astragaloside II were from *Astragalus mongholicus Bunge* [Leguminosae], glycyrrhetic acid, glycyrrhizic acid, licurazide, and isoliquiritigenin came from *Glycyrrhiza uralensis* Fisch. [Leguminosae].

## Discussion

RA is an autoimmune disease that primarily affects synovial joints and is characterized by chronic synovitis, systemic inflammation, and varying degrees of erosion of bone and cartilage. While the cause of RA is unknown, it involves a combination of genetic and environmental factors, potential mechanisms involving the body’s immune system attacking joints, and researchers have focused on exploring the immune mechanisms and molecular networks of RA (Lee and Weinblatt, 2001). Nonsteroidal anti-inflammatory drugs and biologics are currently used to treat RA, but traditional medicine has attracted the attention of doctors and patients in many developing countries because these treatment methods have similar therapeutic goals: anti-inflammatory and pain suppression, slow joint injury, and prevent the development of inflammation-related complications (Moudgil and Berman, 2014; Wang et al., 2021). WTD is a Chinese prescription for rheumatoid arthritis and joint pain which was described in ancient medical writings. As a result, scientific evidence for WTD is accumulating, including clinical efficacy evaluations, characterization of complex components, and molecular mechanism analysis. The RA rat model was effectively duplicated in this work using a variety of assessment methods after injection of Freund’s complete adjuvant in a short period. Through X-ray examination, histopathological analysis, and the detection of inflammatory factors, it was discovered that the model rats’ feet, tails, and spinal joints were gradually swollen and stiff and that there was a severe inflammatory response in the joints of the rats, so the inflammatory response evolved into bone deformation and stiffness due to unregulated, which was the same pathological phenomenon as in previous similar studies (Pan et al., 2017; Dai et al., 2022). Although clinical features of patients with RA cannot be entirely simulated, the current results may explain why rat models of adjuvant-induced arthritis are commonly implemented in drug development.

Studies indicate metabolic disorders in biofluids in RA patients or animal models, covering lipid and glucose metabolism, and amino acid metabolism (Falconer et al., 2018). Untargeted metabolomics with pattern recognition was used to evaluate the metabolites in the serum and urine of RA rats, and 24 different metabolites were detected. Metabolic pathway enrichment results showed crosstalk of three types of pathways dominated by lipid metabolism, of which the important pathway associated with RA rats with an impact value greater than 0.1 was identified (Table 2). Arachidonic acid is a polyunsaturated essential fatty acid located in the body’s fats, the liver, brain, and a range of other organs. Arachidonic acid performs an important role in the structure and function of the body’s cell membranes; it can directly or indirectly synthesize prostaglandins and leukotrienes, and other biologically active substances, and it can cause inflammation in the body by a cascade of arachidonic acid (Charles-Schoeman et al., 2018; Szczuko et al., 2020). The level of arachidonic acid in the model group was abnormal when compared to the control group, which severely disrupted linoleic acid metabolism and arachidonic acid metabolism in lipid metabolism. However, WTD reversed the level of arachidonic acid and may modulate related metabolic pathways, suggesting that this mechanism is both underlying and noteworthy. Lysolecithin is formed by phosphatase A2 hydrolysis of lecithin, and changes in lysolecithin reflect cell renewal and phospholipid consumption (Charles-Schoeman et al., 2018). These compounds are critical in cell proliferation and inflammatory response. WTD has no positive feedback effect on LysoPC(20:5) in the pathway, but it does have a regulatory influence on steroid hormone biosynthesis of lipid metabolism *via* L-palmitoylcarnitine, and high levels of L-palmitoylcarnitine may be detrimental to energy metabolism in mitochondria (Imagawa et al., 1984). Both RA and osteoarthritis patients exhibit anomalies in arginine and proline metabolism in their synovial fluid (Carlson et al., 2018). According to KEGG, hydroxypyruvic acid interfered with glyoxylate and dicarboxylate metabolism, and it might cause cascading fluctuations by acting on arginine and proline metabolism *via* the downstream enzyme 5.3.1.22 (Figure 6C). Unfortunately, WTD only regulates L-arginine and cannot regulate hydroxypyruvic acid.

To discover the Q-markers of WTD, this study employed a chinmedomics strategy to analyze the correlation between the components absorbed into the blood of WTD and the potential biomarkers of RA rats, highlighting the importance of certain components in regulating RA metabolism and markers. Finally, 12 key components were determined as Q-markers for the efficacy of WTD in the treatment of RA. Aconitine, the main active component of *Aconitum carmichaeli* Debeaux [Ranunculaceae], has immunomodulatory properties that might be useful in treating autoimmune diseases, such as rheumatoid arthritis and systemic lupus erythematosus (Li et al., 2017). Ephedrine can mediate toll-like receptor 4 to encourage the production of interleukin 10 and inhibit the secretion of TNF-α to decrease inflammation (Zheng et al., 2012). Quercetin inhibits arthritis mice even stronger than methotrexate (Haleagrahara et al., 2017), and quercetin regulates the enteric nervous system to protect the jejunal inflammation caused by RA (Piovezana et al., 2019). Albiflorin and paeoniflorigenone could modulate the functions and activation of immune cells, decreases inflammatory medium production, and restore abnormal signal pathway (Zhang and Wei, 2020). Astragalin could attenuate synovial inflammation and joint destruction in RA at least partially by restraining the phosphorylation of mitogen-activated protein kinases and the activating of N-terminal kinase/activated protein 1 (Zhang and Wei, 2020). Moreover, kaempferol-3-O-rhamnoside, glycyrrhetic acid, and isoliquiritigenin all have the potential and activity to resist RA (Puchner et al., 2012; Zhu et al., 2012; Ahmed and Abd Elkarim, 2021). With the help of the correlation analysis of chinmedomics, we finally identified 12 Q-markers of WTD, which have a certain degree of anti-inflammatory and immune-regulating biological activity, but it is unclear whether the combination of these compounds has a better anti-RA effect, this needs further verification.

## Conclusion

To summarize, this work used chinmedomics to evaluate the therapeutic effect of WTD on adjuvant-induced arthritic rats and to investigate the Q-markers of WTD. WTD helps alleviate disease states and pathological conditions in inflammatory rats, and chinmedomics as a research tool for TCM effectiveness and pharmacology promotes the discovery of WTD Q-markers and contributes to the theoretical evidence for WTD quality control.

## Data Availability

The original contributions presented in the study are included in the article/Supplementary Material, further inquiries can be directed to the corresponding authors.

## Abbreviations

ESI, electrospray ion source; IL-6, interleukin-6; OPLS-DA, orthogonal partial least squares discriminant analysis; PCA, principal component analysis; PCMS, A plotting of correlation between marker metabolites and serum constituents; Q-markers, quality markers; RA, rheumatoid arthritis; TCM, traditional Chinese medicine; TIC, total ion chromatograms; TNF-α, tumor necrosis factor-alpha; UPLC-Q/TOF-MS, ultraperformance liquid chromatography quadrupole time of flight mass spectrometry; VIP, variable importance in projection value; WTD, wutou decoction.
