# Supplementary material for: High-Throughput Chinmedomics Strategy Discovers the Quality Markers and Mechanisms of Wutou Decoction Therapeutic for Rheumatoid Arthritis
Source: Front Pharmacol. 2022 Apr 12;13:854087. doi: 10.3389/fphar.2022.854087 (PMC9039025; doi:10.3389/fphar.2022.854087)
Supplement: Supplementary file 1 [file DataSheet1.docx]

Supplementary Material

# Supplementary Tables

**Table S1** The results of identification the chemical components in wutou decoction

| **No.** | **Component name** | **m/z** | **Mass error (mDa)** | **R_t_(min)** | **Adducts** | **Formula** | **Fragment** | **Origin** |
| --- | --- | --- | --- | --- | --- | --- | --- | --- |
| 1 | Aconitine | 646.32 | 0.05 | 14.12 | +H | C_34_H_47_NO_11_ | 586.30, 568.29, 554.27 | a |
| 2 | Benzoylmesaconine | 590.30 | 0.66 | 12.70 | +H | C_31_H_43_NO_10_ | 572.29, 540.26, 526.24, 514.24, 105.03, 79.05 | a |
| 3 | Hypaconitine | 616.31 | -0.39 | 16.36 | +H | C_33_H_45_NO_10_ | 584.28, 556.29, 554.26, 105.03 | a |
| 4 | Liquiritigenin | 257.08 | 0.88 | 10.74 | +H | C_15_H_12_O_4_ | 239.07, 163.04, 147.04, 137.06, 123.04, 109.03 | a |
| 5 | Mesaconitine | 632.31 | -0.48 | 15.42 | +H | C_33_H_45_NO_11_ | 572.28, 558.31, 540.26, 123.04 | a |
| 6 | Neoline | 438.29 | 0.72 | 8.74 | +H | C_24_H_39_NO_6_ | 420.27, 406.26, 393.25, 388.25, 374.23, 326.23 | a |
| 7 | 3-Methoxyherbacetin | 317.07 | -0.31 | 11.43 | +H | C_16_H_12_O_7_ | 302.04, 287.06, 169.01 | b |
| 8 | Apigenin-5-rhamnoside | 417.12 | 1.13 | 10.32 | +H | C_21_H_20_O_9_ | 255.07, 149.06, 145.03 | b |
| 9 | Apigenol | 271.06 | 0.48 | 11.95 | +H | C_15_H_10_O_5_ | 253.05, 179.03, 147.04 | b |
| 10 | d-Norpseudoephedrine | 152.11 | 0.61 | 4.68 | +H | C_9_H_13_NO | 134.04, 91.05, 79.05 | b |
| 11 | Kaempferol-3-O-rhamnoside | 433.11 | 0.49 | 10.81 | +H | C_21_H_20_O_10_ | 340.08, 283.12, 195.03 | b |
| 12 | l-Ephedrine | 166.12 | 0.65 | 4.37 | +H | C_10_H_15_NO | 148.11, 132.08, 91.05, 78.05 | b |
| 13 | Leucocyanidin | 307.08 | 2.50 | 1.60 | +H | C_15_H_14_O_7_ | 197.04, 153.02, 125.02 | b |
| 14 | Leucopelargonidin | 291.09 | 0.44 | 5.99 | +H | C_15_H_14_O_6_ | 165.06, 151.04, 139.04, 123.04, 111.04, 95.05 | b |
| 15 | l-Methylephedrine | 180.14 | 1.36 | 5.00 | +H | C_11_H_17_NO | 162.13, 146.10, 135.08, 119.09, 107.05, 93.07 | b |
| 16 | Quercetin | 303.05 | 0.71 | 9.01 | +H | C_15_H_10_O_7_ | 155.04, 125.02, 258.04 | b |
| 17 | Terpinolene | 137.13 | 0.53 | 14.35 | +H | C_10_H_16_ | 121.10, 105.07, 95.09 | b |
| 18 | Tricin | 331.08 | 0.44 | 11.21 | +H | C_17_H_14_O_7_ | 313.07, 301.07, 163.04 | b |
| 19 | Albiflorin | 481.17 | 0.71 | 9.11 | +H | C_23_H_28_O_11_ | 377.15, 319.12, 301.11, 243.09, 215.09, 179.07, 169.09, 107.05 | c |
| 20 | Benzoylpaeoniflorin | 585.20 | -1.06 | 15.77 | +H | C_30_H_32_O_12_ | 319.12, 301.11, 267.09, 249.08, 197.08, 123.04 | c |
| 21 | Gallic acid | 171.03 | 0.57 | 1.76 | +H | C_7_H_6_O_5_ | 153.02, 125.02, 107.01 | c |
| 22 | Paeoniflorigenone | 319.12 | -0.37 | 11.70 | +H | C_17_H_18_O_6_ | 197.08, 184.07, 79.05 | c |
| 23 | Paeonilactone A | 199.10 | 0.99 | 3.45 | +H | C_10_H_14_O_4_ | 181.09, 153.09, 112.05 | c |
| 24 | Paeonilactone B | 197.08 | 0.79 | 9.11 | +H | C_10_H_12_O_4_ | 179.07, 151.08, 125.06 | c |
| 25 | Paeonilactone C | 319.12 | 0.90 | 9.11 | +H | C_17_H_18_O_6_ | 301.11, 243.09, 197.08 | c |
| 26 | Benzoyloxypaeoniflorin | 599.17 | -3.02 | 13.20 | -H | C_30_H_32_O_13_ | 569.17, 445.11, 327.11, 149.05, 137.02, 91.02 | c |
| 27 | Oxypaeoniflorin | 495.15 | 0.64 | 6.39 | -H | C_23_H_28_O_12_ | 465.14, 339.11, 315.09 | c |
| 28 | Lactiflorin | 461.14 | -1.67 | 12.71 | -H | C_23_H_26_O_10_ | 429.12, 339.11, 135.04 | c |
| 29 | Paeonol | 165.05 | -0.89 | 1.38 | -H | C_9_H_10_O_3_ | 150.03, 123.04, 108.02 | c |
| 30 | Kaempferol | 287.06 | 0.13 | 12.62 | +H | C_15_H_10_O_6_ | 271.06, 181.05, 153.02 | b, c, d |
| 31 | 7,2′-Dihydroxy-3′,4′-dimethoxy-isoflavan | 303.12 | 0.72 | 14.33 | +H | C_17_H_18_O_5_ | 269.08, 149.06, 134.04, 133.07, 109.07, 106.04 | d |
| 32 | 8,3′-Dihydroxy-7,4′-dimethoxy-isoflavone | 315.09 | 0.65 | 20.34 | +H | C_17_H_14_O_6_ | 300.06, 271.06, 191.03, 123.04 | d |
| 33 | Astragaline A | 210.08 | 1.87 | 1.08 | +H | C_10_H_11_NO_4_ | 192.07, 126.06, 68.05 | d |
| 34 | Astragaline E | 293.11 | -3.10 | 0.72 | +H | C_14_H_16_N_2_O_5_ | 278.12, 233.09, 180.07, 166.09, 162.05, 150.06, 116.07 | d |
| 35 | Astragaloside Ⅰ | 869.49 | 0.89 | 21.66 | +H | C_45_H_72_O_16_ | 671.42, 487.34, 455.35, 217.07, 199.06, 157.05 | d |
| 36 | Astragaloside Ⅱ | 827.48 | -2.68 | 19.71 | +H | C_43_H_70_O_15_ | 629.04, 175.06, 157.05, 143.11, 127.11, 115.04 | d |
| 37 | Calycosin | 285.08 | 0.71 | 10.78 | +H | C_16_H_12_O_5_ | 270.05, 253.05, 175.04 | d |
| 38 | Calycosin-7-O-β-D-glucopyranoside | 447.13 | 0.44 | 10.78 | +H | C_22_H_22_O_10_ | 343.08, 285.08, 269.04, 253.05, 175.04, 145.03 | d |
| 39 | Isoquercitrin | 465.10 | 0.06 | 10.45 | +H | C_21_H_20_O_12_ | 303.05, 271.06 | d |
| 40 | Isorhamnetin-3-glucoside | 479.12 | -0.19 | 12.57 | +H | C_22_H_22_O_12_ | 317.07, 302.04, 285.08 | d |
| 41 | Kaempferide-4′-methyl ether-3-glucoside | 463.12 | 0.31 | 11.16 | +H | C_22_H_22_O_11_ | 301.07, 283.06, 93.03 | d |
| 42 | Agroastragaloside Ⅳ | 987.51 | -3.93 | 20.52 | -H | C_49_H_80_O_20_ | 941.51, 807.42, 175.02 | d |
| 43 | 7-Hydroxy-3',4'dimethoxy-isoflavan-5', 2′-di-O-β-D-glucoside | 641.21 | -1.52 | 9.42 | -H | C_29_H_38_O_16_ | 593.15, 297.08, 161.04 | d |
| 44 | Odoratin-7-O-β-D-glucoside | 431.13 | -1.71 | 9.62 | -H | C_22_H_24_O_9_ | 309.10, 299.09, 161.05 | d |
| 45 | 2′-Hydroxy-3′,4′-dimethoxy-isoflavan-7-O-β-D-glucoside | 463.16 | -0.87 | 14.29 | -H | C_23_H_28_O_10_ | 301.11, 286.08, 153.05 | d |
| 46 | 8,2′-Dihydroxy-7,4′-dimethoxy-isoflavan | 301.11 | -0.85 | 14.29 | -H | C_17_H_18_O_5_ | 286.03, 271.06, 164.05 | d |
| 47 | 3,4-Dihydroxycinnamic acid | 179.03 | -0.13 | 6.53 | -H | C_9_H_8_O_4_ | 135.04, 133.03, 91.02 | d |
| 48 | 18β-Glycyrrhetic acid (Glycyrrhetic acid) | 471.35 | -0.39 | 18.97 | +H | C_30_H_46_O_4_ | 453.34, 435.33, 407.33, 383.26, 331.23, 221.15 | e |
| 49 | 7-Methoxycoumarin | 177.06 | 0.44 | 6.12 | +H | C_10_H_8_O_3_ | 145.03, 134.04, 106.04 | e |
| 50 | Deoxyglabrolide | 455.35 | -0.38 | 14.88 | +H | C_30_H_46_O_3_ | 437.34, 301.22, 247.17 | e |
| 51 | Glabrolide | 469.33 | 0.80 | 16.95 | +H | C_30_H_44_O_4_ | 451.32, 423.33, 383.26, 315.20, 261.15, 219.14 | e |
| 52 | Glycyrrhizic acid | 823.41 | -0.65 | 18.96 | +H | C_42_H_62_O_16_ | 647.38, 471.35, 453.34, 425.34, 383.26, 263.17, 221.15, 167.11 | e |
| 53 | Isoglabrolide | 469.33 | -0.64 | 17.36 | +H | C_30_H_44_O_4_ | 451.32, 261.15, 233.15, 219.14, 151.08 | e |
| 54 | Isoliquiritin | 419.13 | 1.08 | 12.77 | +H | C_21_H_22_O_9_ | 299.09, 283.06, 239.07, 163.04, 137.02, 119.05 | e |
| 55 | Licurazide | 551.18 | 1.18 | 12.77 | +H | C_26_H_30_O_13_ | 419.13, 283.06, 257.08, 239.07, 163.04, 137.02 | e |
| 56 | Liquiritin | 419.13 | 0.67 | 12.97 | +H | C_21_H_22_O_9_ | 313.07, 285.08, 257.08, 237.06, 163.04, 137.06 | e |
| 57 | Isoliquiritigenin | 255.07 | -0.01 | 12.92 | -H | C_15_H_12_O_4_ | 237.06, 161.02, 148.05, 145.03, 135.01, 119.05 | e |
| 58 | Neoliquiritin | 417.12 | 0.35 | 8.51 | -H | C_21_H_22_O_9_ | 297.08, 235.05, 132.04 | e |
| 59 | Licochalcone B | 285.08 | -1.75 | 13.13 | -H | C_16_H_14_O_5_ | 270.05, 255.06, 175.04 | e |
| 60 | Formononetin | 269.08 | 1.05 | 13.16 | +H | C_16_H_12_O_4_ | 253.05, 237.06, 163.04, 136.02, 133.06, 107.05 | e, d |

| 61 |
| --- |

Note: a: *Aconitum carmichaeli* Debeaux [Ranunculaceae], b: *Ephedra sinica* Stapf [Ephedraceae], c: *Paeonia lactiflora* Pall. [Ranunculaceae], d: *Astragalus mongholicus Bunge* [Leguminosae], e: *Glycyrrhiza uralensis* Fisch. [Leguminosae].

**Table S2** Identification of the components in serum after oral administration of wutou decoction solution

| **No.** | **Component name** | **m/z** | **Mass error (mDa)** | **R_t_ (min)** | **Adducts** | **Formula** | **Fragment** |
| --- | --- | --- | --- | --- | --- | --- | --- |
| 1 | 18β-Glycyrrhetic acid+2x(+O)+SO_3_ | 583.30 | 2.80 | 10.89 | +H | C_30_H_46_O_9_S | 568.27, 335.12, 535.27 |
| 2 | Aconitine-C_7_H_4_O-H_2_+C_2_H_2_O | 580.28 | 2.00 | 10.68 | -H | C_29_H_43_NO_11_ | 580.28, 297.19, 562.27 |
| 3 | Aconitine-H_2_ | 644.31 | 1.50 | 10.91 | +H | C_34_H_45_NO_11_ | 297.19, 89.06, 87.04 |
| 4 | Albiflorin | 479.16 | 1.80 | 9.11 | -H | C_23_H_28_O_11_ | 445.15, 242.08, 224.07 |
| 5 | Albiflorin-C_17_H_16_O_5_+H_2_ | 181.07 | -2.80 | 1.31 | -H | C_6_H_14_O_6_ | 180.07, 162.05, 136.08 |
| 6 | Astragaline A | 208.06 | -1.40 | 1.08 | -H | C_10_H_11_NO_4_ | 194.08, 108.05 |
| 7 | Astragaloside Ⅰ+O+H_2_+C_6_H_8_O_6_ | 1061.52 | 1.40 | 10.78 | -H | C_51_H_82_O_23_ | 1012.49, 888.40, 396.20 |
| 8 | Astragaloside Ⅱ-C_6_H_10_O_5_+2x(+O)+SO_3_ | 777.38 | 2.90 | 11.13 | +H | C_37_H_60_O_15_S | 746.36, 717.35, 587.33 |
| 9 | Glycyrrhizic acid | 823.41 | 0.90 | 18.96 | +H | C_42_H_62_O_16_ | 647.38, 471.35, 453.34, 425.34, 383.26, 263.17, 221.15, 167.11 |
| 10 | Glycyrrhizic acid-C_6_H_8_O_6_+2x(+O)+C_2_H_2_O | 719.37 | 4.30 | 11.48 | -H | C_38_H_56_O_13_ | 319.19 |
| 11 | Hypaconitine-C_7_H_4_O_2_+2x(+H_2_)+C_2_H_2_O | 542.34 | 4.00 | 14.65 | +H | C_28_H_47_NO_9_ | 89.06, 73.03 |
| 12 | Hypaconitine-C_7_H_4_O_2_+H_2_+C_6_H_8_O_6_ | 674.34 | -2.50 | 9.20 | +H | C_32_H_51_NO_14_ | 656.33, 468.29 |
| 13 | Isoliquiritigenin | 257.08 | 1.00 | 12.92 | +H | C_15_H_12_O_4_ | 147.04, 137.02, 119.05 |
| 14 | Kaempferol-3-O-rhamnoside | 431.10 | -0.20 | 10.81 | -H | C_21_H_20_O_10_ | 268.04, 129.02, 87.01 |
| 15 | l-Ephedrine | 164.11 | 4.37 | 4.37 | -H | C_10_H_15_NO | 91.06, 77.04 |
| 16 | l-Ephedrine-CH_2_+2x(-H_2_)+C_2_H_2_O | 188.07 | -0.70 | 13.27 | -H | C_11_H_11_NO_2_ | 188.07, 168.05, 133.06 |
| 17 | l-Ephedrine-CH_3_N+H_2_ | 139.11 | 0.50 | 21.07 | +H | C_9_H_14_O | 119.09 |
| 18 | l-Ephedrine-CH_3_N-H_2_+SO_3_ | 213.02 | -1.00 | 12.73 | -H | C_9_H_10_O_4_S | 213.02, 133.07 |
| 19 | Licurazide | 549.16 | -1.00 | 12.77 | -H | C_26_H_30_O_13_ | 175.02, 147.04, 113.02 |
| 20 | l-Methylephedrine | 180.14 | 0.30 | 5.00 | +H | C_11_H_17_NO | 162.13, 146.10, 135.08, 119.09, 107.05, 93.07 |
| 21 | l-Methylephedrine-H_2_+C_6_H_8_O_6_ | 354.16 | 0.40 | 3.86 | +H | C_17_H_23_NO_7_ | 164.11, 149.08, 133.07 |
| 22 | Mesaconitine+H_2_ | 634.32 | -1.50 | 10.91 | +H | C_33_H_47_NO_11_ | 481.23, 287.21, 89.06 |
| 23 | Neoliquiritin | 417.12 | -1.20 | 8.51 | -H | C_21_H_22_O_9_ | 297.08, 235.05, 132.04 |
| 24 | Paeoniflorigenone | 319.12 | 3.80 | 11.70 | +H | C_17_H_18_O_6_ | 197.08, 184.07, 79.05 |
| 25 | Paeoniflorigenone-C_10_H_12_O_5_+H_2_+SO_3_ | 187.01 | -1.00 | 7.94 | -H | C_7_H_8_O_4_S | 187.01, 107.05 |
| 26 | Paeonilactone A | 197.08 | -1.10 | 3.45 | -H | C_10_H_14_O_4_ | 153.09, 111.04, 85.03 |
| 27 | Quercetin+H_2_+SO_3_ | 383.01 | -1.70 | 8.97 | -H | C_15_H_12_O_10_S | 366.00, 350.10, 184.95 |
| 28 | Tricin | 329.07 | 1.80 | 11.21 | -H | C_17_H_14_O_7_ | 313.07, 284.03, 148.99 |

# 2 Supplementary Figures

**
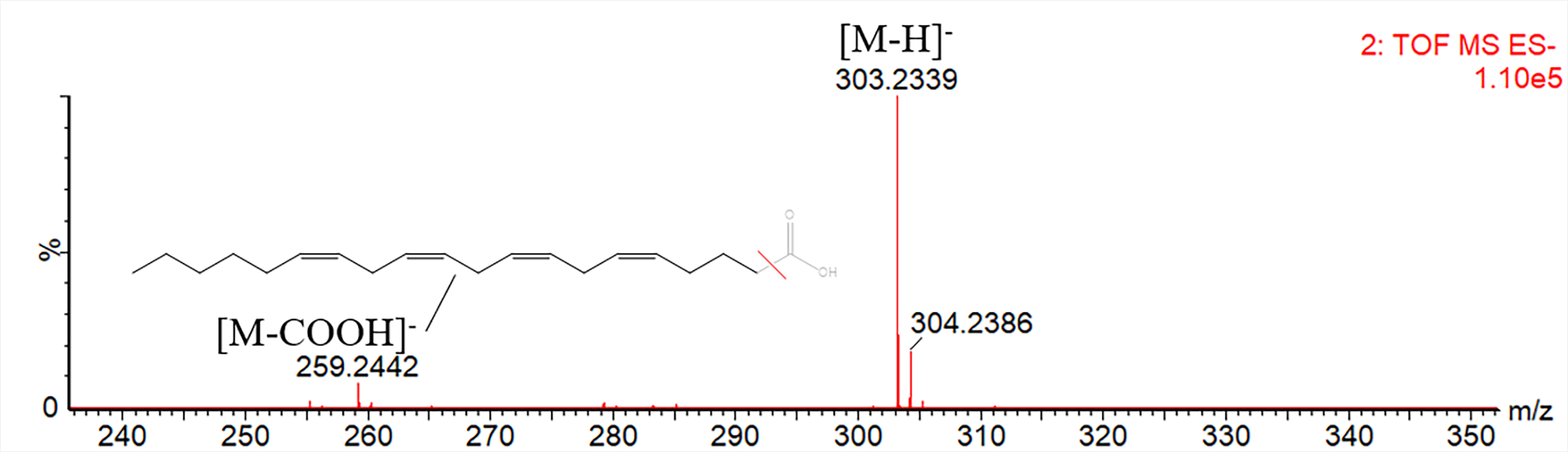
**

**Figure S1.** Identification of arachidonic acid by ion extraction and fragmentation analysis of MS^E^ data.

**
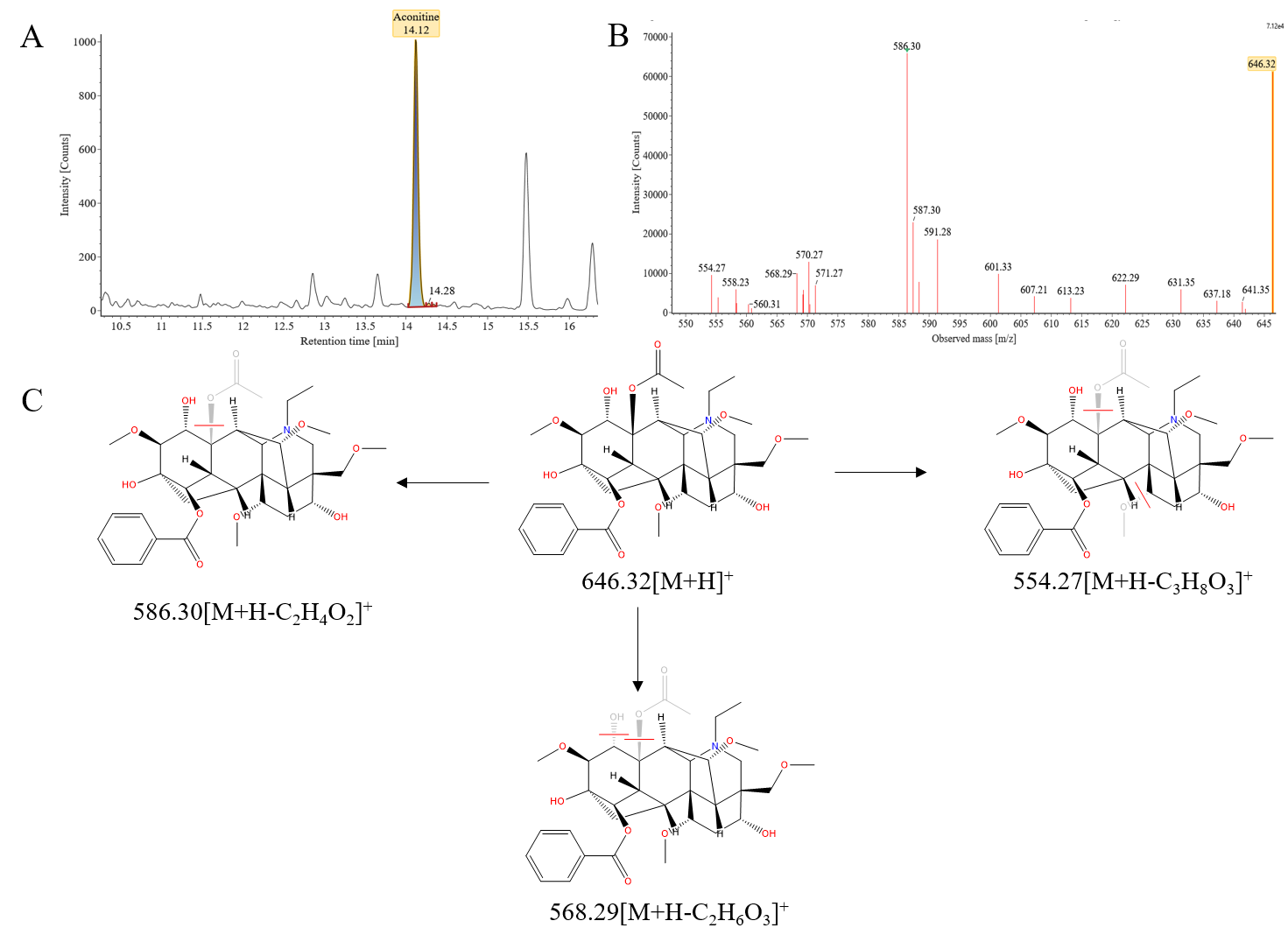
**

**Figure S2.** MS^E^ format data are combined with UNIFI software to identify the process of aconitine. **(A)** Chromatographic peak of the extracted aconitine **(B)** MS/MS information of aconitine under high energy **(C)** Cleavage pathway of aconitine.
